# Gallbladder Torsion: A Diagnostic Challenge

**DOI:** 10.1155/2014/902814

**Published:** 2014-05-08

**Authors:** Sharon Gabizon, Kimberley Bradshaw, Eshwarshanker Jeyarajan, Rafid Alzubaidy, Victor Liew

**Affiliations:** Department of Surgery, Robina Hospital, 2 Bayberry Lane, Robina, QLD 4226, Australia

## Abstract

80-year-old female presented with clinical findings suggestive of acute cholecystitis. Intraoperatively we discovered a dusky gallbladder with gangrenous patches and gallbladder torsion with 270 degrees clockwise rotation along the longitudinal axis. Gallbladder torsion is a rare cause of acute cholecystitis with less than 500 cases published in the literature. Gallbladder torsion should be included in the list of differential diagnoses in patients suspected of having acute cholecystitis especially when there are inconsistencies between clinical features and imaging. It is worth noting that 3-dimensional reconstructed CT may be useful in preoperative diagnosis of gallbladder torsion.

## 1. Introduction

Gallbladder torsion is a rare presentation of gallbladder pathology. Features are generally consistent with acute cholecystitis and the diagnosis is usually made intraoperatively. Further research is needed to assess the benefits of imaging modalities preoperatively, specifically 3-dimensional CT reconstruction in gallbladder pathology. If gallbladder torsion is suspected or diagnosed, prompt surgical intervention is required.

## 2. Case Presentation

**Figure 1:**
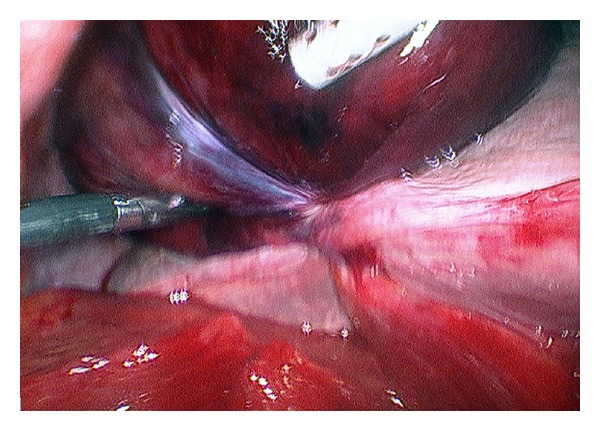
Obstructed, haemorrhagic gallbladder and clockwise 270 degrees rotation around the gallbladder longitudinal axis.

**Figure 2:**
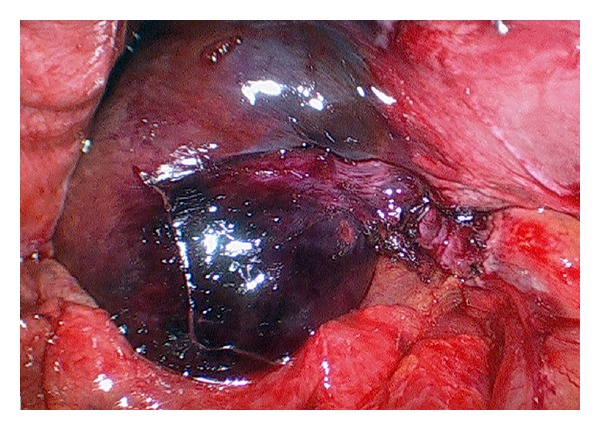
Gallbladder seen following untwisting of the mesentery.

An 80 year old female presented with clinical findings suggestive of acute cholecystitis. Her pain was unusual such that it was constant in nature, rated as 8/10 in her right upper quadrant and there was no clinical improvement over 24 hours. An abdominal ultrasound scan was performed, which reported small mobile gallstones, a distended thick walled gallbladder with surrounding pericholecystic fluid and a dilated common bile duct. There was no impacted stone seen in the cystic duct or Hartmann's pouch. Hence a request for CT was made and again it confirmed no calculous obstruction. The patient was managed with intravenous antibiotics initially but had worsening pain, fever to 38.6°C, white cell count of 14 × 10^9^/L and a C-reactive protein of 81 before a decision was made to proceed to emergency laparoscopic cholecystectomy the following day. Intra-operatively we discovered a dusky gallbladder with gangrenous patches and torsion of the gallbladder with 270 degrees clockwise rotation along the longitudinal axis (Figures 1 and 2). Histology confirmed gallbladder haemorrhage, necrosis and mucosal sloughing, consistent with ischaemia. Post-operatively we reviewed the CT again with the radiologist and a 3-dimensional volume reconstruction was requested. In retrospect it was clearly evident that there was a transition point at the neck of the gallbladder and torsion.

## 3. Discussion

Pathophysiology of acute calculous cholecystitis is mostly due to impacted stone in the Hartmann's pouch or cystic duct with gallbladder outflow obstruction, biliary stasis and bacterial overgrowth. In contrast, acalculous cholecystitis is related to poor perfusion of the gallbladder, acute inflammation with necrosis, bile stasis, bacterial overgrowth and secondary infection. This case demonstrated that gallbladder torsion is another possible mechanism for acute gangrenous cholecystitis which is rarely suspected pre-operatively, yet may require immediate surgery and if not treated promptly could potentially lead to septic or fatal complications.

The history and physical examination findings on presentation were suggestive of acute cholecystitis. At no stage could we differentiate between torsion and gallstone cholecystitis from the history and examination findings alone. CT abdomen is not commonly requested to make the diagnosis of acute cholecystitis except in selected cases with obstructive jaundice where choledocholithiasis, head of pancreas malignancy or cholangiocarcinoma are suspected. In this case we did not suspect torsion but the severity of the pain was atypical, not consistent with and could not be explained by the ultrasound findings, hence further imaging was requested.

Torsion of the gallbladder, also known as “floating gallbladder” was first reported by Wendel in 1898 [1] and since then there has been approximately 500 cases reported in the literature [2]. It has been reported to occur 1 in 365,520 hospital admissions and is more common in females in their seventh and eighth decades of life [3, 4]. The aetiology is still not certain although it is thought that a long gallbladder mesentery with minimal or no attachment to the liver facilitates the longitudinal axis of the gallbladder to twist on its vascular root resulting in ischaemia, necrosis and perforation [4].

**Figure 3:**
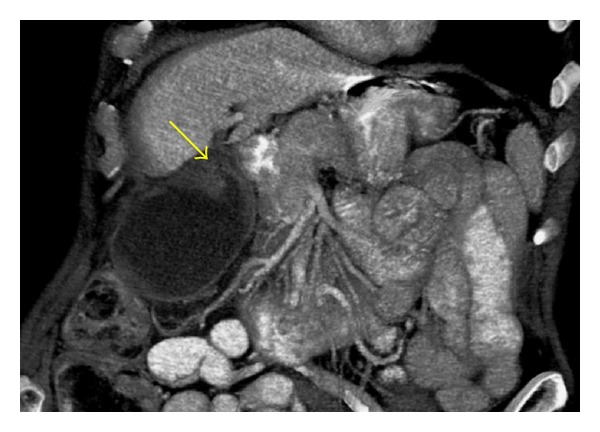
3-Dimensional volume reconstruction CT abdomen to confirm a distended gallbladder with a clear transition point (arrow), pericholecystic fluid, inflammatory stranding and enhancement of the gallbladder wall.

To make the diagnosis of gallbladder torsion is difficult, with less than 10% of cases reported pre-operatively in the literature [5]. Kitagawa et al. published the diagnostic imaging criteria necessary for identifying gallbladder torsion which include fluid between the gallbladder and the gallbladder fossa of the liver, a horizontal rather than vertical long axis of the gallbladder, enhancing cystic duct and oedema associated with a thickened gallbladder wall [6]. To date, there are no published cases of gallbladder torsion diagnosed from 3-dimensional CT reconstruction, which would have assisted us to establish a diagnosis pre-operatively (Figure 3) and proceed to surgery without delay.

This case demonstrates the importance of understanding the patho-physiology of acute cholecystitis. Having a high index of suspicion for other causes of acute cholecystitis in the absence of simple gall stone obstruction, or when investigation results do not correlate with the clinical presentation, gallbladder torsion should still be considered even if it is an extremely rare entity.
